# Variable Non-Gaussian Transport of Nanoplastic on Supported Lipid Bilayers in Saline Conditions

**DOI:** 10.1021/acs.jpclett.4c00806

**Published:** 2024-05-14

**Authors:** Diyali Sil, Edin Osmanbasic, Sasthi Charan Mandal, Atanu Acharya, Chayan Dutta

**Affiliations:** † Department of Chemistry, Georgia State University, Atlanta, Georgia 30303, United States; ‡ Department of Chemistry, Syracuse University, Syracuse, New York 13244, United States; § BioInspired Syracuse, Syracuse University, Syracuse, New York 13244, United States

## Abstract

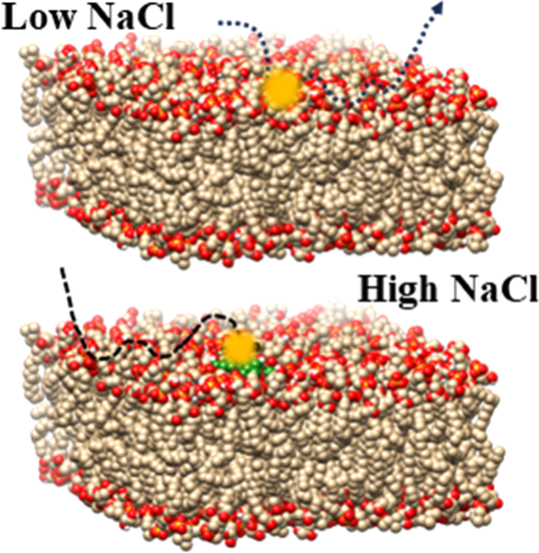

Nanoplastic–lipid interaction is vital to understanding the nanoscale mechanism of plastic adsorption and aggregation on a lipid membrane surface. However, a single-particle mechanistic picture of the nanoplastic transport process on a lipid surface remains unclear. Here, we report a salt-dependent non-Gaussian transport mechanism of polystyrene particles on a supported 1-palmitoyl-2-oleoyl-glycero-3-phosphocholine (POPC) lipid bilayer surface. Particle stickiness on the POPC surface increases with salt concentration, where the particles stay longer at the surface and diffuse to shorter distances. Additionally, a non-Gaussian diffusion state dominates the transport process at high salt concentrations. Our current study provides insight into the transport mechanism of polystyrene (PS) particles on supported lipid membranes, which is essential to understanding fundamental questions regarding the adsorption mechanisms of nanoplastics on lipid surfaces.

Plastic pollution is a global concern,^1^ and understanding plastic polymer–bilayer membrane interaction is key to uncovering the effects of nano pollutants such as plastic nanoparticles (PNPs).^2,3^ Lipid membranes consist of a bilayer with hydrophilic heads and hydrophobic hydrocarbon tails, resulting in a surface that is hydrophilic and a core that is hydrophobic. A balance between hydrophobic and hydrophilic forces dominates the extent of polymer interaction with lipid membranes.^4−6^ Polymer adsorption on lipid surfaces leads to lipid reorganization and domain formation with increased rigidity, inducing local heterogeneity at the surface.^7^ Coarse-grained molecular dynamics simulations also predicted that polystyrene (PS) nanoparticles could adsorb to the lipid membrane surface, penetrate the hydrophilic surface and accumulate at the hydrophobic core, and disrupt the membrane structure.^8,9^ Insertion of polyethylene nanoparticle inside the 1-palmitoyl-2-oleoyl-glycero-3-phosphocholine (POPC) lipid bilayer resulted in hydrocarbon chain reorganization increasing the bilayer thickness while reducing the area per lipid.^9^ Molecular dynamics (MD) simulation reveals that PS particles affect the dynamics, architecture, and cooperativity of dipalmitoyl-PC (DPPC) lipid bilayers.^10^ Rossi et al. highlight that PS chains preferentially partition to the liquid-disordered (Ld) domain in phase-separated lipid membranes, indicating that PS chains have a major impact on the dynamics and lateral organization of multicomponent lipid bilayer consisting of unsaturated phosphatidylcholine (PC) lipids (dilinoleyl-PC, DUPC), saturated PC lipids (dipalmitoyl-PC, DPPC), and cholesterol (CHOL).^8^ Hence, understanding the mechanism of PNP transport at the single-particle level is required for understanding the true effect of PNP interaction on biosystems.

Desorption-mediated random walk and confined diffusion are typically observed in single-particle studies on soft polymer surfaces.^11−15^ Non-Gaussian transport has been observed in various soft-matter systems where the mean square displacement (MSD) is proportional to time but the displacement distribution probability shows convoluted distribution as opposed to a Gaussian curve.^16,17^ Colloidal particles can follow non-Gaussian type of transport where at short displacements particles show confined motion and at long displacements Brownian diffusion prevails.^17^ Transport under a salt environment plays a crucial role in biological systems, and a nanoscale understanding of salt effects on the nanoplastic transport mechanism is lacking.

Here, we provide a detailed single-particle investigation of nanoplastic transport on a supported lipid bilayer surface under variable saline conditions. We also used equilibrium MD simulations and steered molecular dynamics (SMD) simulations to understand the relative number density and positions of specific ions correlated with single-particle measurements. Our results showed two distinct transport modes for carboxy-functionalized PS particles on the POPC lipid surface, where a Fickian but non-Gaussian transport mechanism was observed for various salt conditions. A charge-dipole-dependent transport model was proposed at physiologically relevant salt concentrations where particles are adsorbed on the surface at higher salt and exhibit confined diffusion. These studies show the importance of a single-particle understanding of the nanoscale effects of PNPs to uncover the nanoplastics–membrane interaction mechanisms.

Two transport modes are observed for PS bead transport on the surface of the POPC bilayer surface. The schematic of the experimental system is presented in Figure 1A, showing a supported lipid bilayer of POPC. Carboxy-functionalized PS beads at a very dilute concentration were flowed over the supported lipid bilayer (SLB) surface in a microfluidic flow chamber (see the Materials and Methods section in the Supporting Information for details of the sample preparation and instrumentation) and measured using a total internal reflection fluorescence (TIRF) microscope (Figure S1). Our analysis with the localization-based single-particle tracking algorithm (Troika^18^) revealed two different types of trajectories. Qualitatively, we can group these trajectories into Brownian diffusion modes, where particles show longer displacements, and confined diffusion, with particles showing short displacements. Figure 1B shows two representative trajectories of each type.

**Figure 1:**
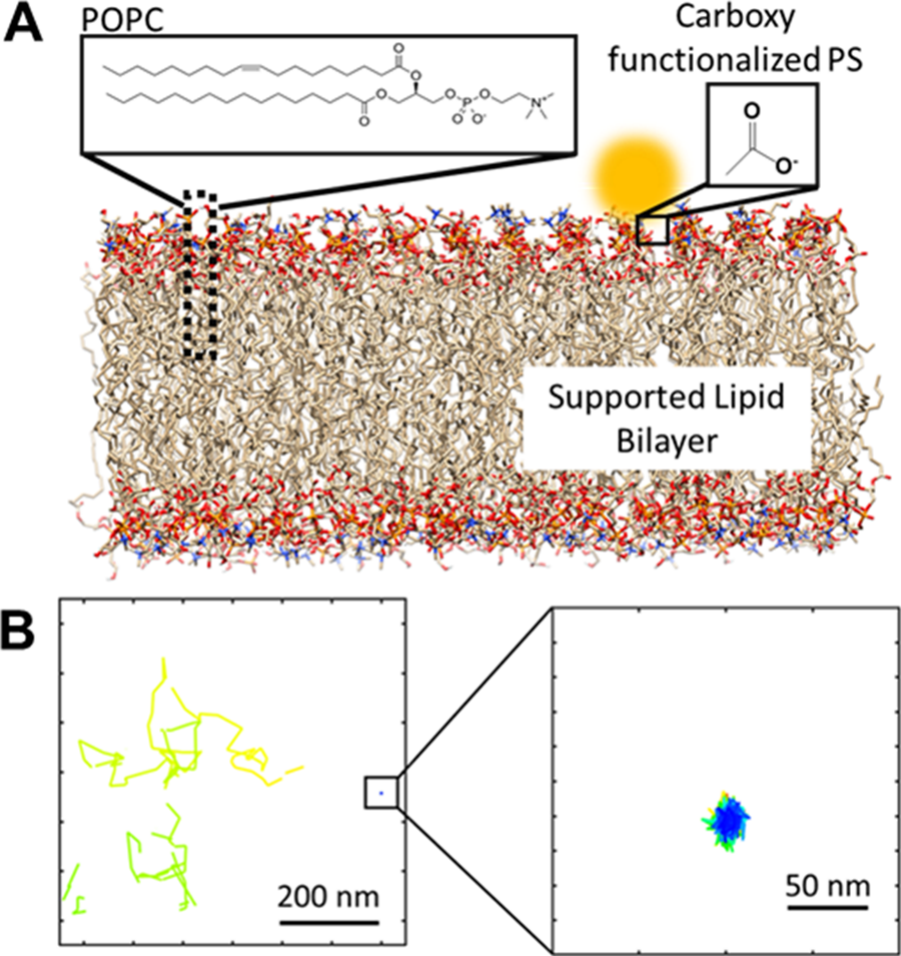
Two types of transport modes for PS on POPC bilayers. (A) An experimental model system showing a carboxy functionalized PS bead (100 nm) on a supported POPC bilayer surface. (B) Representative trajectory showing a random diffusion with longer displacement and confined diffusion for PS beads.

The salt environment increases PS bead adsorption on the POPC surface, and PS particles accumulate on the lipid surface under high salt conditions. To understand the effect of salt, we performed a salt concentration-dependent study by varying the NaCl concentrations between 10, 100, and 1000 μM in HEPES buffer. Figure 2A,B shows representative frames from the raw movies at 10 and 1000 μM salt, respectively. At 10 μM NaCl, we observe almost no difference in particle adsorption with that under only buffer conditions (Figure S2). However, with increasing salt concentration, more PS particles interacted with the surface and more particles were stuck to the surface.

**Figure 2:**
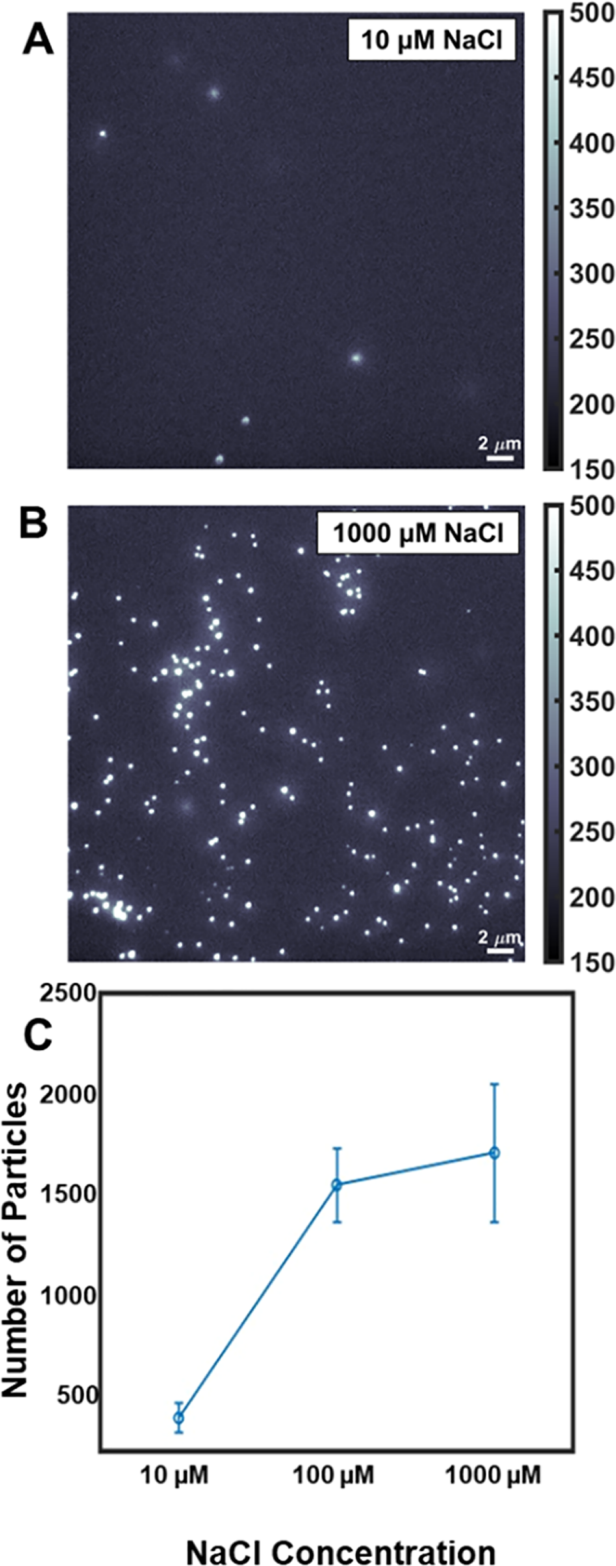
A higher number of particles are adsorbed on the POPC supported lipid bilayer surface at high salt concentrations. (A and B) Representative raw data showing PS beads on a supported POPC bilayer at 10 uM and 1000 uM NaCl, respectively. (C) Total number of PS particles on the lipid bilayer surface as a function of salt (NaCl) concentration. Here the number of particles at each concentration is calculated as an average from at least 10 different data sets, and the error bar represents the standard deviations from all measurements.

We quantified the number of particles that interacted with the POPC surface by calculating the total number of particle trajectories from at least 10 different data sets, as presented in Figure 2C (Table S1). The number of particles at each concentration represents the average trajectories for the data sets collected at equal time intervals from the same region of interest (ROI) at the lipid surface. The error bar represents the standard deviation at each condition.

It is evident from Figure 2C that a higher number of particles are adsorbed on the surface, showing greater affinity for the PS particles for the POPC surface at high salt concentrations. We also compared the number of particle trajectories under no salt conditions and after washing (washing with buffer) all salt from the surface (Table S1) to find that the number of detected trajectories after wash conditions is always higher than initial conditions.

Particle displacement is dependent on the salt concentration. PS mobility on the POPC surface is quantified using the single-frame displacement (SFD) distributions that show the mobility of the particles between two consecutive frames and are plotted as histograms of all displacement events. We determined the relative population distribution using a Markov chain Monte Carlo (MCMC) algorithm and obtained the relative populations observed in the SFD distributions.^13,19^ Representative SFD distributions for three salt conditions are plotted in Figure 3A (see Figure S3 and Table S2 for MCMC fitting parameters). We observe two distinct populations of displacements and denote them as long and short, respectively. These two population distributions change as a function of salt concentration, which is indicative of the variable local heterogeneity in nanoplastic–membrane interaction. We note that a single-PS trajectory can contribute to both populations if a PS particle undergoes random diffusion followed by periodic immobilization or confined diffusion.^20^

**Figure 3:**
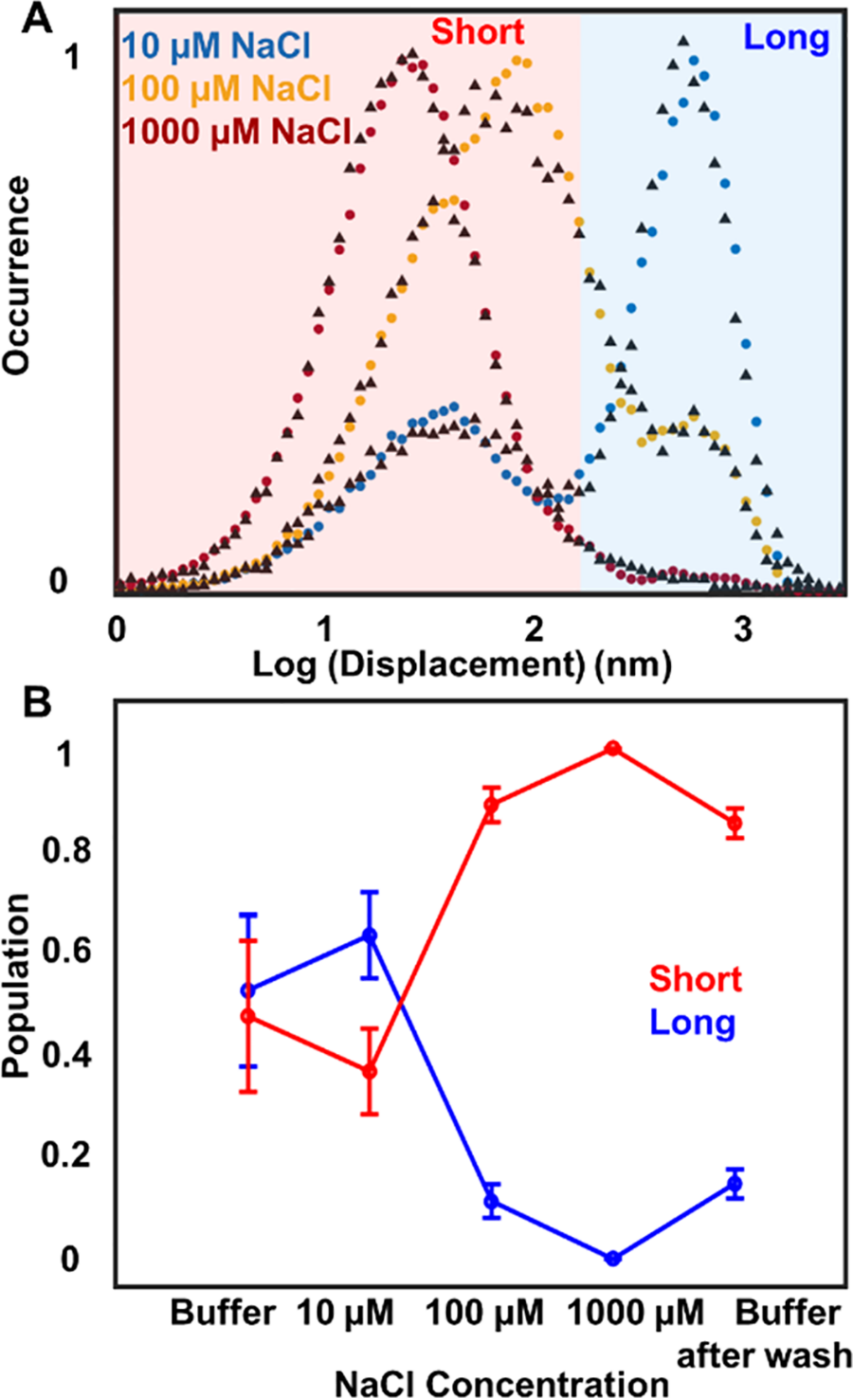
Displacement modulation for long and short displacements of PS particles from single-particle trajectories at varying salt concentrations. (A) Single-frame displacement distribution for carboxy-functionalized PS beads on the supported POPC surface under varying NaCl salt concentrations from 10 to 1000 μM. Two different populations of displacements are observed in all conditions. Each single-frame displacement distribution curve (dotted lines with filled circles) represents at least 3000 single-particle events. Dotted lines with black triangles represent the MCMC approximation of the distribution. (B) Long and short populations at each concentration obtained from MCMC analysis are plotted as a function of NaCl. As concentration increases, the long displacement is decreased, while the short population increases. Here each point is the average of 10 different data sets, and the error bar represents the standard deviation.

The long displacement population decreases with salt concentration, while the short displacement population increases, as shown in Figure 3B. Here, we plotted the populations obtained from the MCMC analysis at 10, 100, and 1000 μM NaCl. We also show the population changes in HEPES buffer and after washing conditions. The relative changes between buffer and 10 μM conditions are within the error range of our experimental analysis. However, after washing all salt from the surface, the displacements do not compare with that of the initial buffer conditions. We think the irreversibility observed in particle mobility even after washing all salt is due to an irreversible change in membrane fluctuation^16^ due to salt exposure.

Particles stay longer on the surface at high salt concentrations. Surface residence times (SRTs) corresponding to the time a particular trajectory is active on the surface are calculated and plotted as a cumulative probability distribution, *P*(*t* > t_0_). These cumulative probability distribution curves (Figure 4A) were fitted with three exponentials assuming a first-order kinetic model, and the corresponding weighted average time scales are calculated at each condition (Figure S4 and Table S3 for SRT fitting details) with the following equations: *P*(*t* > *t*_0_) =  and τ_avg_ = . Here,  is the population of the *i*th component with the corresponding time scale *τ*_*i*_. The average surface residence time (*τ*_Avg_) spent by the PS particles on the lipid surface increases with an increasing salt concentration (Figure 4B). We also observed that *τ*_Avg_ for the PS beads do not revert to comparable time scales when the sample is washed thoroughly using buffer (Table S3) demonstrating the irreversibility in particle interactions.

**Figure 4:**
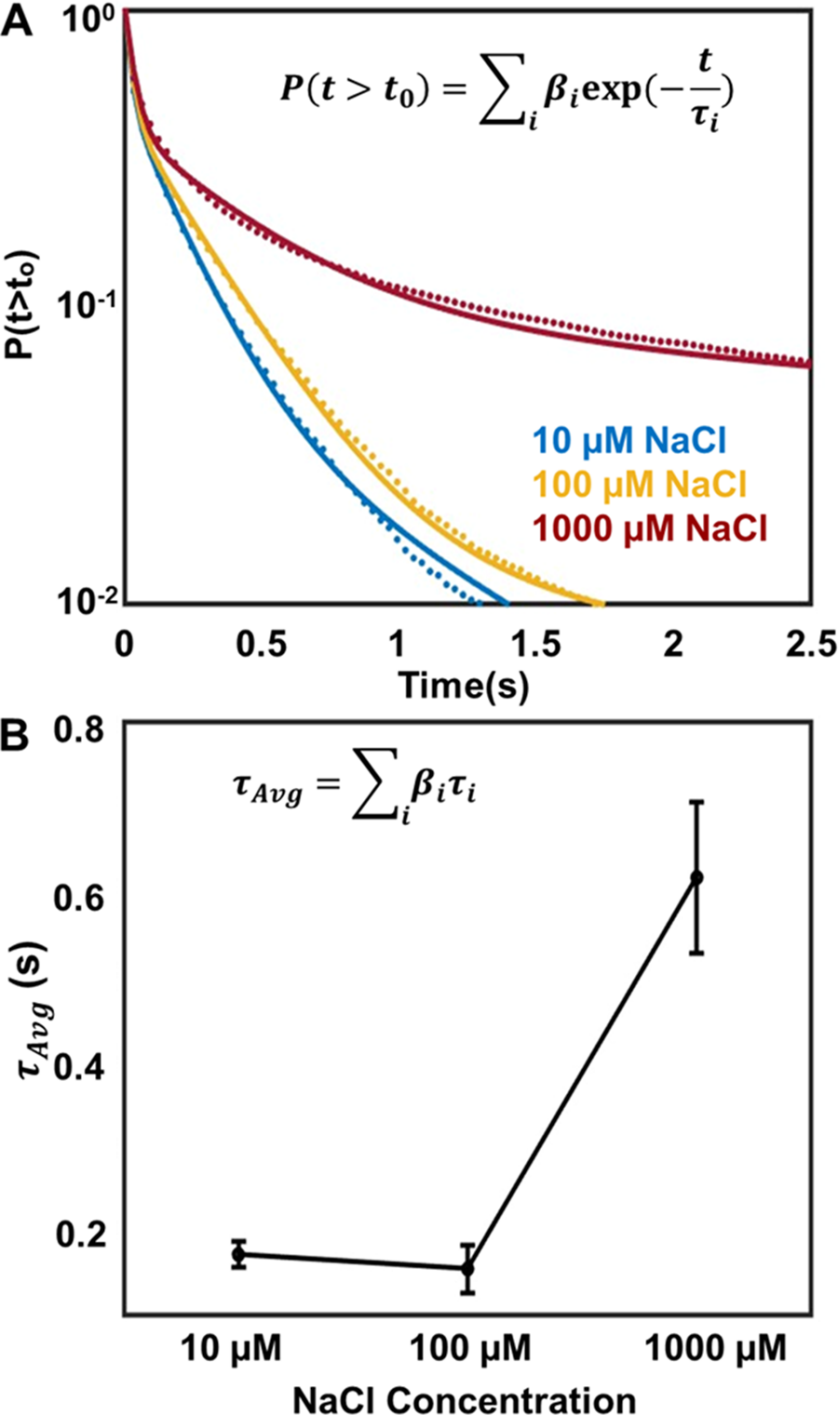
Particles stay longer at the lipid surface at high NaCl concentration. (A) Cumulative SRT probability distributions are plotted at 10, 100, and 1000 μM NaCl concentrations. The dotted lines represent the fittings at each concentration. (B) Average surface residence times () as a function of NaCl concentration. Each data point is an average of 10 different data sets, and the error bars represent the standard deviations in each measurement.

Distributions of particle displacements calculated using the self-part of the van Hove correlation function show heterogeneity in PS bead trajectories. Displacement distributions were calculated at the shortest time lag (0.03 s) for all salt concentrations using the following equation:
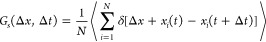
1where *Δx* represents the displacement along the coordinate axis, *Δt* represents time lag, and *N* is the total number of displacements at a specific time lag. Qualitatively, the distributions could be divided into two regions, as presented in Figure 5A showing the displacements at 10, 100, and 1000 μM NaCl concentrations. We find a sharp central peak (displacement < ∼0.2 μM) and a shoulder region at higher displacements that often is broad and heavy-tailed for all concentrations. Such distributions are consistent with our raw data, where we see single particles with 2D diffusion followed by confinement or confined (stuck) particles diffusing away from the surface after some period of confinement (representative raw movies are attached in the Supporting Information). Distributions at different salt concentrations show similar plots with two characteristic regions; however, the broad Gaussian region is reduced at high salt concentrations, showing fewer long displacements consistent with the raw data. The broad Gaussian gradually disappears, and an exponential tail dominates at higher salt concentrations. The time evolution of the van Hove distribution points to a heterogeneous transport mechanism of the PS beads under our experimental conditions.

**Figure 5:**
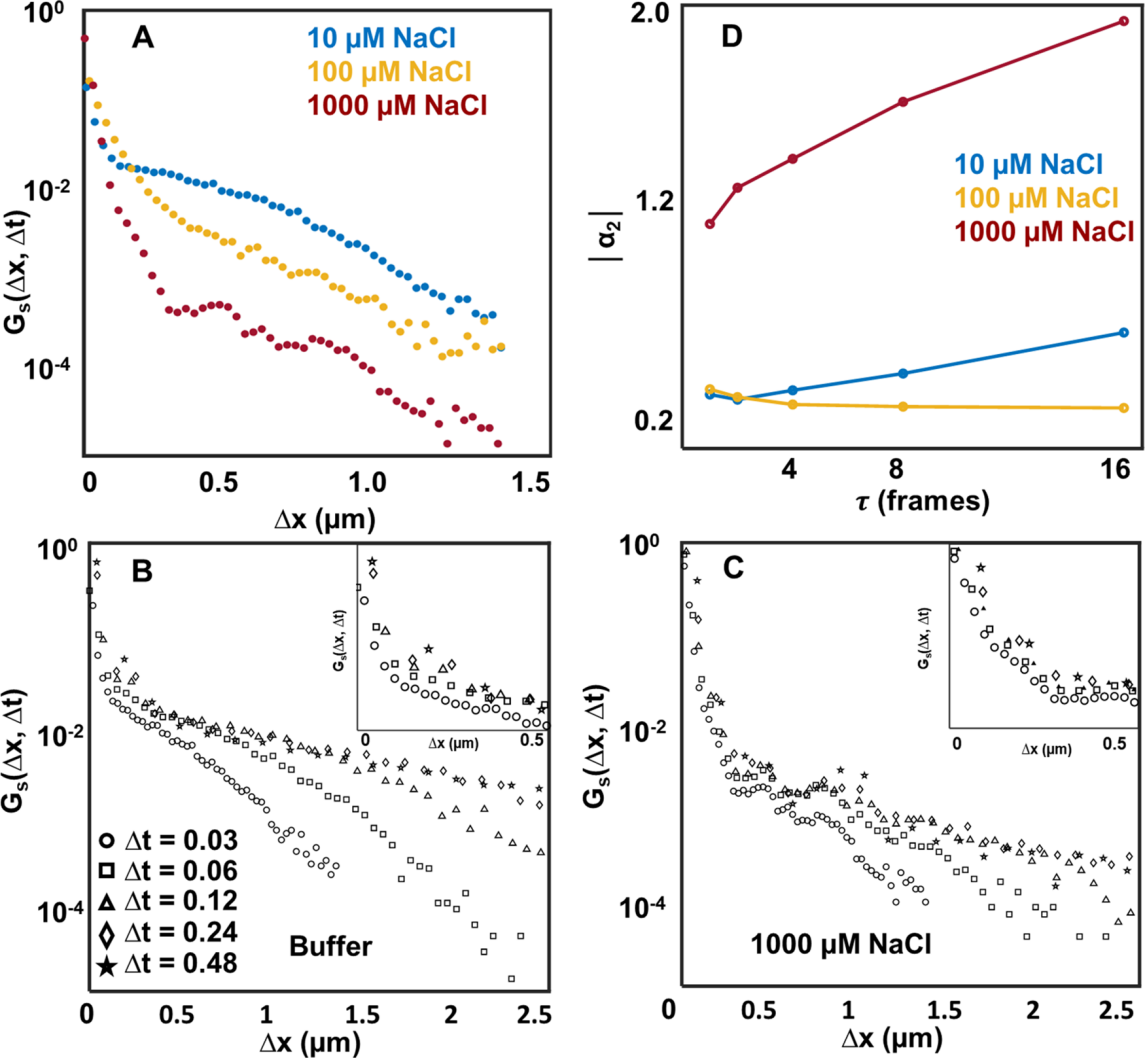
Non-Gaussian diffusion at high NaCl concentration. (A) Self-part of the van Hove distribution for representative particle trajectories at 10, 100, and 1000 μM NaCl concentration. A central peak at short displacements (<0.2 μm) and a broad distribution (>0.5 μm) are observed at all conditions. (B) Self-part of the van Hove distributions at only buffer and (C) 1000 μM NaCl concentrations at different time lags. In both cases, the central peak broadens with time lag (data presented at insets for clarity), showing confined diffusion. The broad Gaussian peak at the buffer condition broadens with time, typical for surface-mediated transport processes. Also, the long-tailed distribution for the 1000 μM NaCl broadens with time. (D) The non-Gaussian parameter (α_2_) calculated at different time lags shows increasing non-Gaussian behavior for all conditions.

Variance of the central peak changes with time lag,^21^ which shows apparent particle displacements during strong confinement or immobility. We performed time-lag-dependent distribution analysis to understand the time evolution of the two regions. In buffer conditions, the width of the central peak increases from ∼0.1 to ∼0.5 μm (Figure 5B), whereas the increase of the central peak in all other salt conditions is lower. Figure 5C presents the time evolution of the distribution for 1000 μM NaCl, showing a small gradual increase in the central peak (inset).

Broad Gaussian distributions in the shoulder region, representing displacements with short flights, are mainly observed (Figure 5A) in the buffer and 10 and 100 μM NaCl. Such diffusion processes are observed on homogeneous surfaces, which are interpreted as lacking strong chemical confinement and are characteristic of desorption-mediated continuous time random walk processes.^22,23^ Time lag analysis of the distributions (Figure S5) shows that the Gaussian tail regions at longer displacements for 10 and 100 μM remain Gaussian, but the width of the distribution increases as expected for Brownian diffusion processes. For 1000 μM, we see that the exponential tail dominates at higher time lag values. Here, we note that the localization precision of our instrument is around 10 nm in both directions (Figure S6). Hence, any confined trajectories could not be resolved below the localization limit.

The observed diffusion of PS particles on the lipid bilayer surface is Fickian but non-Gaussian. Fickian diffusion follows that the MSD scales with time according to a power law and shows a Gaussian distribution of displacements.^17^ However, in soft-matter systems, non-Gaussian transport has been observed where the displacement distributions often have multiple convoluted Gaussian and exponential distributions, while the MSD still scales with time. MSD plots with a time lag (Figure S7 and Table S5) showed a straight line, and a power law fitting produced the average diffusion coefficient between 1 and 3 μm^2^ s^–1^, consistent with previously reported fast diffusion at lipid surfaces. A non-Gaussian parameter, *α*_2_, defined as α_2_(τ) = , can quantify the deviation from the expected Gaussian model, where  is the displacement of a particle at a time τ and  and  describe the forth and second moment of the probability distribution, respectively.^24−26^ For non-Gaussian transport, α_2_ is nonzero and it increases with time for heterogeneous particle transport. Figure 5D (Table S4) shows that the α_2_ value is greater than 0 for all concentrations at all time lags and it increases rapidly with time for 1000 μM NaCl, suggesting strong heterogeneity due to the confined motion of PS particles on the POPC surface.

Based on our experimental observation of PS transport on a POPC lipid surface, we propose a salt-dependent transport model, as presented in Scheme 1. POPC is a zwitterionic lipid with a charged headgroup. The Dimova group has shown previously that alkali metal cations preferentially adsorb to the POPC membrane, increasing the net surface potential of the POPC membranes using isothermal titration calorimetry and zeta potential measurements.^27^ Metal ion adsorption leads to a slightly positive charge at the surface, and the POPC surface is not neutral at higher salt concentrations. Weak interaction between the negatively charged PS beads and charge neutral POPC surface prevails at low NaCl concentrations, where the PS beads, when closer to the surface, exhibit longer transport distances, shorter residence times, and mostly continuous time random walk type diffusion.

**Scheme 1:**
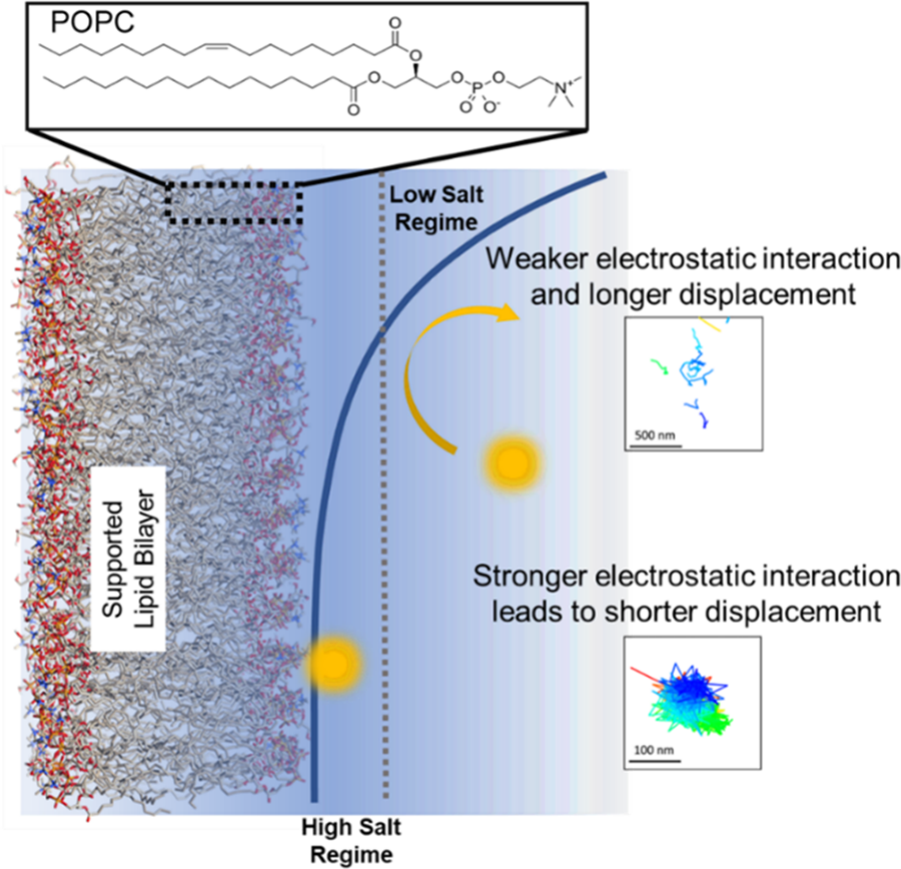
Salt-Dependent Transport Model of Carboxy Modified PS on Supported POPC Surface Debye length decreases with increasing salt concentration, so at low ionic strength there is lower electrostatic interaction between the negatively charged PS beads and the zwitterionic surface head groups of the POPC lipids leading to longer displacement and lower surface adsorption. At higher salt concentration, stronger electrostatic interactions lead to higher adsorption, shorter displacement, and confined diffusion.

We calculated the number density of membrane headgroups and quantified the specific interactions with Na^+^ and Cl^–^ ions to show that Na^+^ ions preferentially interact with the phosphate head of the POPC (Figure 6). First, we discuss the number density of membrane headgroups,  and  (Figure 6A), and Na^+^ and Cl^–^ ions (Figure 6B) along the *z*-axis (membrane normal) from the last 800 ns of the equilibrium simulations. The position of the peaks for both the headgroups appears nearly at the same *z* positions in both the leaflets. This is because the broadening of the density profile extends approximately 20 Å, whereas the average *z* distance (calculated from 800 ns equilibrium MD simulations) between the phosphorus atom (P) of the  group and the nitrogen atom (N) of the  moiety is only about 1.4 Å. Similarly, we calculate the density of Na^+^ and Cl^–^ ions, shown in Figure 6B. The peak positions for Na^+^ are much closer to the membrane surface compared to the peak positions for Cl^–^. The difference between the two peak positions for Na^+^ and Cl^–^ ions is about 10 Å, while the average *z* distance between the two head groups is 1.4 Å. This indicates that Na^+^ ions exhibit a preference for residing in closer proximity to the membrane surface than Cl^–^ ions. Figure 6C highlights the frequent –Na^+^ interaction, where a representative snapshot is selected from the equilibrium simulations. Similarly, the sparse  bound Cl^–^ ions are shown in Figure 6D.

**Figure 6:**
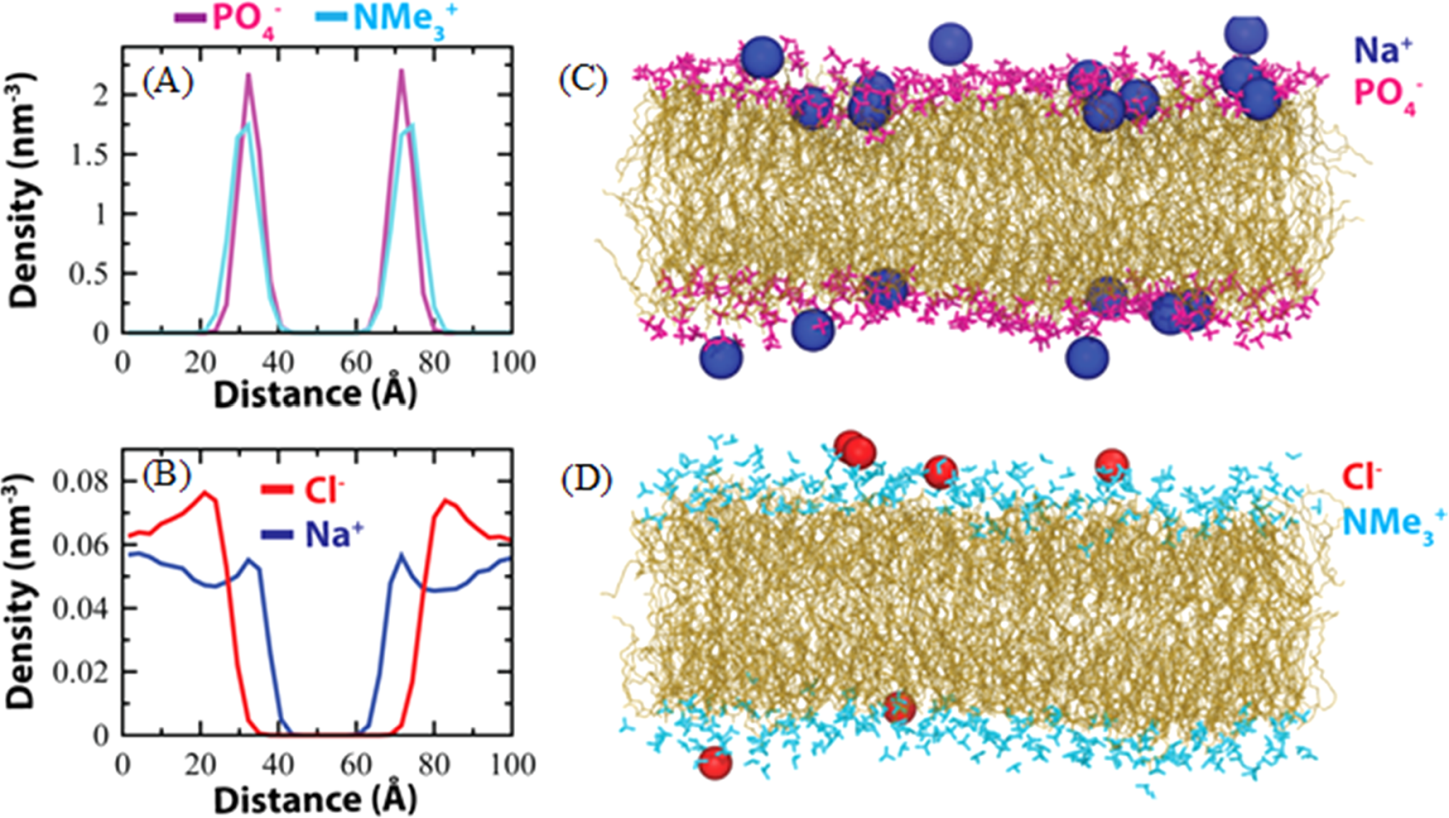
(A) The number density profile of the lipid headgroups,  (magenta) and  (cyan) along the *z*-axis of the simulation box. (B) The density profile for Na^+^(blue) and Cl^–^ (red) ions. Representative snapshots show Na^+^ ions are bound to the  groups (C) and Cl^–^ ions bound to the  groups (D). The lipid tails are shown in yellow.

Na^+^ ions tend to localize near the negatively charged phosphate groups of the phosphatidylcholine (PC) lipids, exposing the positively charged quaternary amine groups to the surface.^28^ We also calculate the average number of Na^+^ and Cl^–^ ions that are within 5.0 Å of the  groups and the  groups, respectively, shown in Table S6. We observe that a higher number of Na^+^ ions interact with  compared to the number of Cl^–^ interactions with . Density profiles for the headgroups and ions do not show any observable difference in the presence of PS polymers from SMD simulations (Figures S10 and S11). Similar trends were observed in 100 ns simulations in windows of interest from the umbrella sampling (Figure S11). A strong electrostatic interaction between the negatively charged PS beads and the positively charged amines leads to shorter displacement and longer residence times at the surface. Lipid layers can swell in the presence of salt, and the membrane bending rigidity increases, as previous theoretical and experimental studies suggested. However, these effects are smaller at our experimental NaCl concentrations.^29,30^ We imaged the prepared SLB samples using Nile red (NR) as previously reported by Sharonov et al.^31^ NR does not fluoresce in water but is fluorescent in the hydrophobic core of the lipid bilayer. We find that we have a uniform coverage of NR over our membrane surface (Figure S8), and the uniform coverage of NR is maintained after keeping the lipid membranes under 10 μM and 1000 μM NaCl concentrations over 30 and 90 min (Figure S9). We conclude that the overall membrane structure does not change in our experimental conditions; hence, the observed transport properties of PS particles are not influenced by any salt-induced nanoscale alteration to the membrane.

Lateral diffusion of phospholipid in a supported bilayer has been shown to have a fast and slow diffusion component when polymers adsorb on the surface.^17^ Polymers, when adsorbed on a lipid surface, create nanodomains with slower lipid diffusion that is dependent on the adsorbate particle size.^32−34^ Negatively charged nanoparticles can induce local gelation at the fluid neutral lipid surface, and as a consequence, the gelled patches at the contact region of lipid-nanoparticle will be stiffer.^7^ Our single-particle diffusion studies of negatively charged PS particles on the POPC membrane indicate that the particle adsorption is higher at high NaCl with longer surface residence times for the PS particles, while the particles diffuse to shorter distances following non-Gaussian distribution. Particle adsorption induces local stiffness at the lipid surface and a higher confined diffusion.

In conclusion, we report the salt-dependent heterogeneous non-Gaussian transport of model nanoplastic on the zwitterionic lipid surface. Due to the high surface-to-volume ratio, nanoplastics are prone to absorb and penetrate cell membranes and influence diverse biological processes.^35^ As the salt environment plays an important role in biological systems, understanding how PNPs adhere to the membrane surface and become adsorbed under varying salt concentrations is important.^36^ Our studies lay the foundation for a nanoscale understanding of nanoplastic–membrane interactions with broader implications in membrane biophysics during environmental pollutant exposure.

## Supporting Information Available

Materials and Methods; chemicals for sample preparation; supported lipid bilayer (SLB) preparation; microfluidic device preparation for single-particle measurements; total internal reflection fluorescence imaging; single-particle tracking (SPT) analysis; quantifying the number of adsorbed particles on the POPC surface at various conditions; total number of particles at the surface; control experiment on glass coverslip; Markov chain Monte Carlo (MCMC) analysis on single-frame displacement distributions for all conditions; SFD fitting parameters; two-component vs three-component fittings for SRT distributions; van Hove correlation analysis; time-dependent van Hove graphs; non-Gaussian parameter analysis; localization precision; mean square displacement analysis; SLB membrane imaging with Nile Red (NR) dye; SLB membrane imaging after salt exposure to the membrane; system preparation for molecular dynamics simulations; details of the MD simulation; calculation of potential of mean force (PMF); calculation of number density (PDF)
