# Supplementary material for: Variable Non-Gaussian Transport of Nanoplastic on Supported Lipid Bilayers in Saline Conditions
Source: J Phys Chem Lett. 2024 May 14;15(20):5428–35. doi: 10.1021/acs.jpclett.4c00806 (PMC11129298; doi:10.1021/acs.jpclett.4c00806)
Supplement: Supplementary file 1 — jz4c00806_si_001.pdf [file jz4c00806_si_001.pdf]

Variable Non-Gaussian Transport of Nanoplastic on Supported Lipid Bilayers in Saline Conditions

Diyali Sil[1], Edin Osmanbasic[1], Sasthi Charan Mandal,[2] Atanu Acharya,[2,3] Chayan Dutta[1,*]

[1]Department of Chemistry, Georgia State University, Atlanta 30303, United States

[2]Department of Chemistry, Syracuse University, Syracuse, New York 13244, United States

[3]BioInspired Syracuse, Syracuse University, Syracuse, New York 13244, United States

*** Corresponding Author**:

Chayan Dutta, Email: cdutta@gsu.edu

Table of Contents:

Experimental Section 3

Materials and Methods

1. Chemicals for sample preparation 3
2. Supported Lipid Bilayer (SLB) membrane preparation 3
3. Microfluidic device preparation for single particle measurements 3
4. Total Internal Reflection Fluorescence Imaging 4

Data Analysis 4

1. Single Particle Tracking (SPT) analysis 4
2. Quantifying the number of adsorbed particles on POPC surface at various conditions 5
3. Markov Chain Monte Carlo (MCMC) analysis on single frame displacement distributions 7
4. Single frame displacement (SFG) fitting parameters 8
5. Surface Residence Time (SRT) fitting 9
6. Van-Hove Correlation analysis 11
7. Non-Gaussian parameter analysis 12
8. Localization precision of our analysis 13
9. Mean Square Displacement analysis 14
10. SLB membrane imaging with Nile Red (NR) dye 15
11. SLB membrane imaging after salt exposure to the membrane 16
12. System preparation for molecular dynamics simulations 17
13. Details of the MD simulation 18
14. Calculation of potential of mean force (PMF) 19
15. Calculation of number density. 20
16. References 16

Experimental Section

Materials and methods

Chemicals for sample preparation

Palmitoyl-2-oleoyl-sn-glycero-3-phosphocholine (POPC) (Avanti Polar Lipids), HEPES buffer (pH 7.3) (Quality Biological), chloroform (HPLC Stab, VWR CHEMICALS) and sodium chloride (NaCl) (99%, Beantown Chemical) were purchased and used as received. The HEPES buffer was of initial concentration 1M and was diluted to 20 mM with Millipore water (Avidity Science). POPC lipid was dissolved in chloroform to make a concentration of 25 mg/ml and diluted to 5 mg/ml with 20 mM HEPES buffer for small unilamellar vesicles (SUV) preparation.

Supported lipid bilayer (SLB) preparation

Supported lipid bilayer was formed through the vesicle fusion process.[1, 2] A solution of POPC lipid (Avanti Polar Lipids) in HEPES buffer having a concentration of 5 mg/ml was extruded through a polycarbonate membrane of pore size 100 nm. The extruded SUV solution (~ 50 μL) was then injected into the microfluidic chamber on the hydrophilic surface of the plasma cleaned coverslip. After 90 minutes, the chamber was washed with copious amount of HEPES buffer (2 times, at leaset 500 μL) and then the supported lipid bilayer (SLB) was ready for single-particle imaging.

Microfluidic device preparation for single particle measurements

We prepared the SLB of POPC lipid on a plasma cleaned cover glass and a microfluidic sample chamber having an inlet as well as an outlet. A very dilute solution (2.2×10^8 particles/ml) of carboxy modified PS bead solution is passed through the microfluidic with a speed of 200 nL/min

and the transport of PS beads is measured using a total internal reflection fluorescence (TIRF) microscope (**Figure S1**).

Total Internal Reflection Fluorescence Imaging (TIRF)

A wide-field TIRF microscope (Nikon) was used to capture single-particle fluorescence images of PS beads with video rate imaging. Using a 561 nm laser for excitation, the beam was focused onto the sample surface using a 100X NA 1.49 oil-immersion objective. Data acquisition was conducted over a 512×512-pixel area on the sample, with fluorescence emission collected by an EMCCD camera (Andor, iXon 897). The camera operated using Andor Solis software in frame transfer mode, with an integration time set to 30 ms.

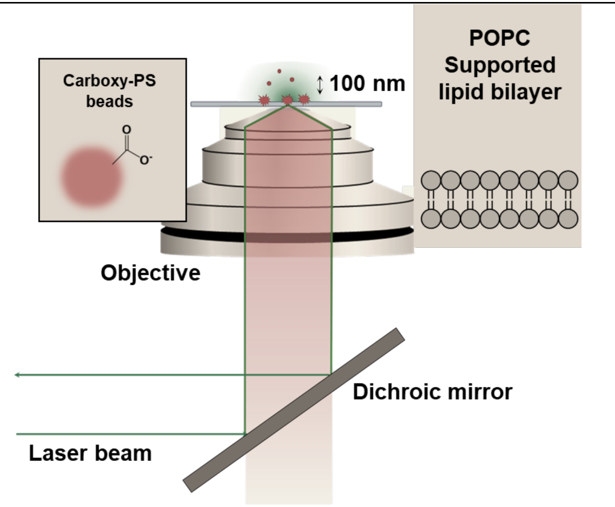

Figure S1: Schematic of the TIRF microscope and the microfluidic sample.

Data Analysis

Single Particle Tracking (SPT) analysis

We have used a localization based single particle tracking algorithm (Troika[3]) to identify, localize, and track individual PS particles over multiple frames. Troika performs three key sequential steps to analyze the raw video files; first the signal-to-noise is enhanced by multiplication with a pixel averaging matrix, then each particle on each frames are identified by local intensity maximum and radial symmetry fitting,[4] and finally localized particle positions are linked using a nearest neighbor algorithm to generate particle trajectories at different conditions. Here we note that, we have used

a first frame filtering Matlab script to reject any stuck particle that have been identified on the first frame to reduce redundancy and bias in our data analysis. We performed the first frame filtering as we have observed already stuck particles on the surface for some salt conditions. Statistical analysis is performed on all the trajectories at different conditions as explained in the following sections.

Quantifying the number of adsorbed particles on the POPC surface at various conditions

We counted the total number of trajectories under each condition for multiple data sets and presented them in **Table S1**. The average and standard deviation are calculated in each case and compared with the blank coverslip. We find that there are a few particles that we could identify on black coverslip and under buffer conditions, however, the number of stuck particles under these conditions are negligible. However, with increasing salt concentration, total number of individual particle trajectories as well as the number of stuck particles increased.

Total number of particles at the surface

	On Coverslip	Buffer	10 µM	100 µM	1000 µM	After Wash
**Data Set 1**	250	178	293	1200	926	350
**Data Set 2**	162	135	287	1342	1403	346
**Data Set 3**	122	147	370	1398	1441	510
**Data Set 4**	132	199	260	1466	1598	422
**Data Set 5**	197	218	365	1487	1720	503
**Data Set 6**	175	225	361	1534	1838	617
**Data Set 7**	166	178	394	1711	1903	538
**Data Set 8**	132	231	432	1672	1950	530
**Data Set 9**	226	180	518	1779	2055	528
**Data Set 10**	152	280	424	1773	2131	432
**Average (count)**	**171**	**197**	**370**	**1536**	**1697**	**478**
**Standard Deviation**	**40**	**41**	**74**	**185**	**346**	**83**

Table S1: Number of particles on the surface. All data collected from the same region of the lipid bilayer surface at different NaCl salt concentrations. A gradual increase in the number PS particles interacting with the lipid surface is confirmed from the average number of particles.

Control experiment on glass coverslip

Interaction of blank glass coverslip with the PS particles are negligible as compared to the SLB at both low and high salt concentrations. We showed some representative frames from raw data on a blank coverslip and compared to the SLB surface images in buffer conditions and at 1000 µM salt concentration. More numbers of active particles are observed at high salt concentration.

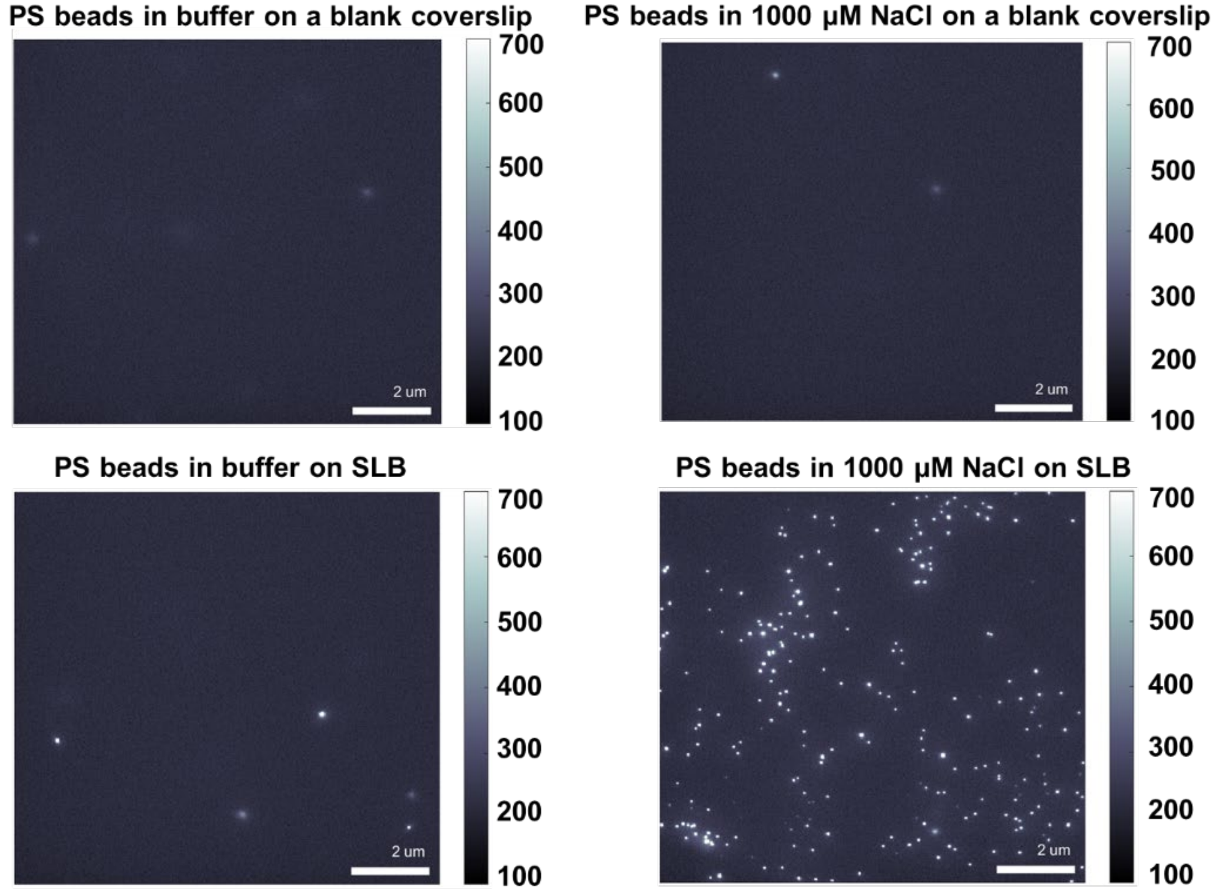

Figure S2: Control experiment of PS bead with blank coverslip (No SLB) in buffer and 1000 µM NaCl solution. Representative frames from the raw data have been plotted for different conditions. The scale bar is 2 µm.

Markov Chain Monte Carlo (MCMC) analysis on single frame displacement (SFD) distributions for all conditions

SFD distributions and and MCMC analysis[5] for all trajectories are presented in **Figure S2**. Generally, we observe two populations in the SFD distribution and the average and standard deviations for each populations are presented in **Table S1**. For 1000 µM NaCl, we only observe a short population in SFD with a broader full width at half maxima. We also see a small shoulder at large displacements, however, the MCMC analysis was unable to resolve this shoulder peak. Hence we approximated that the short population have the 100% contribution to overall displacements albeit with a broader distribution, consistent with our raw data.

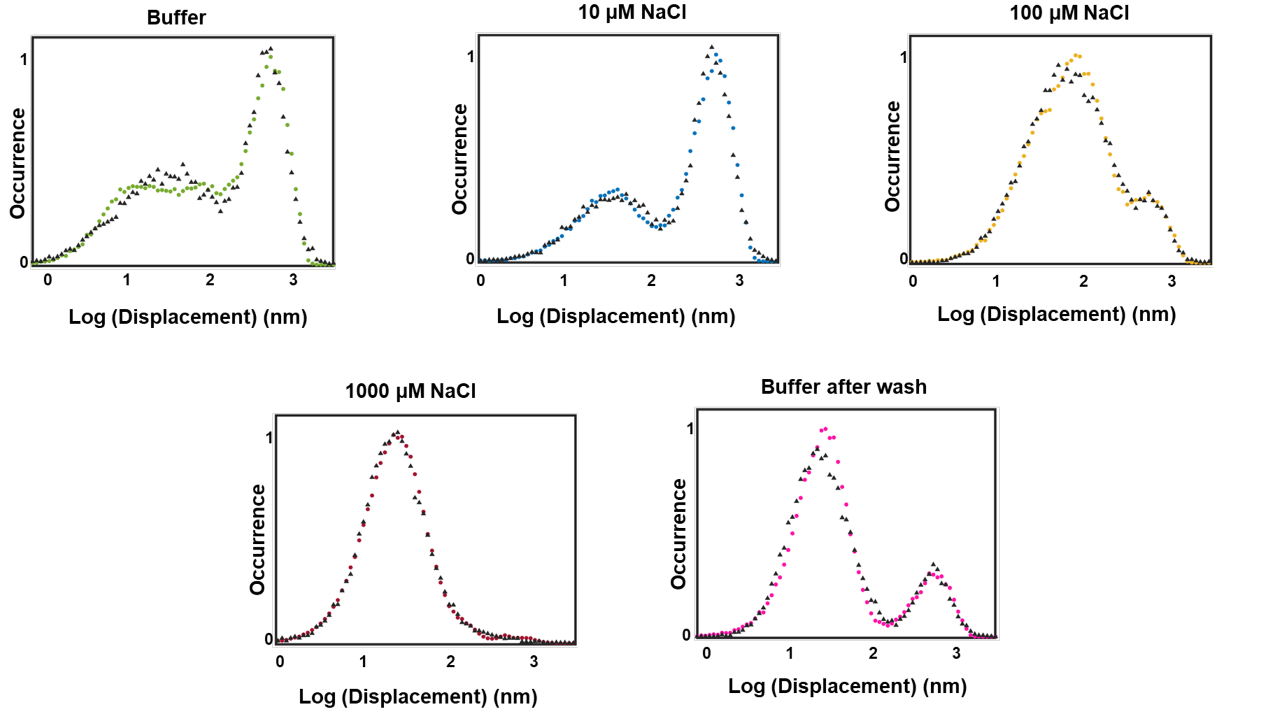

Figure S3: SFD distribution of carboxy functionalized PS beads on the supported POPC lipid bilayer surface in buffer (green),10 µM NaCl (blue), 100 µM NaCl (yellow), 1000 µM NaCl (dark red) and buffer after washing (pink) condition.

SFD fitting parameters

	Short Displacement		Long Displacement	
	Average	Standard Deviation	Average	Standard Deviation
**Buffer Only**	0.475	0.1485	0.525	0.1485
**10 µM**	0.3667	0.0839	0.6333	0.0839
**100 µM**	0.8889	0.0337	0.1119	0.0329
**1000 µM**	1	—	0	—
**Buffer After Wash**	0.83533	0.0289	0.1467	0.0289

Table S2: Average and standard deviations of short and long displacement populations calculated from Gaussian sampling of single frame displacement graphs.

Surface Residence Time (SRT) fitting

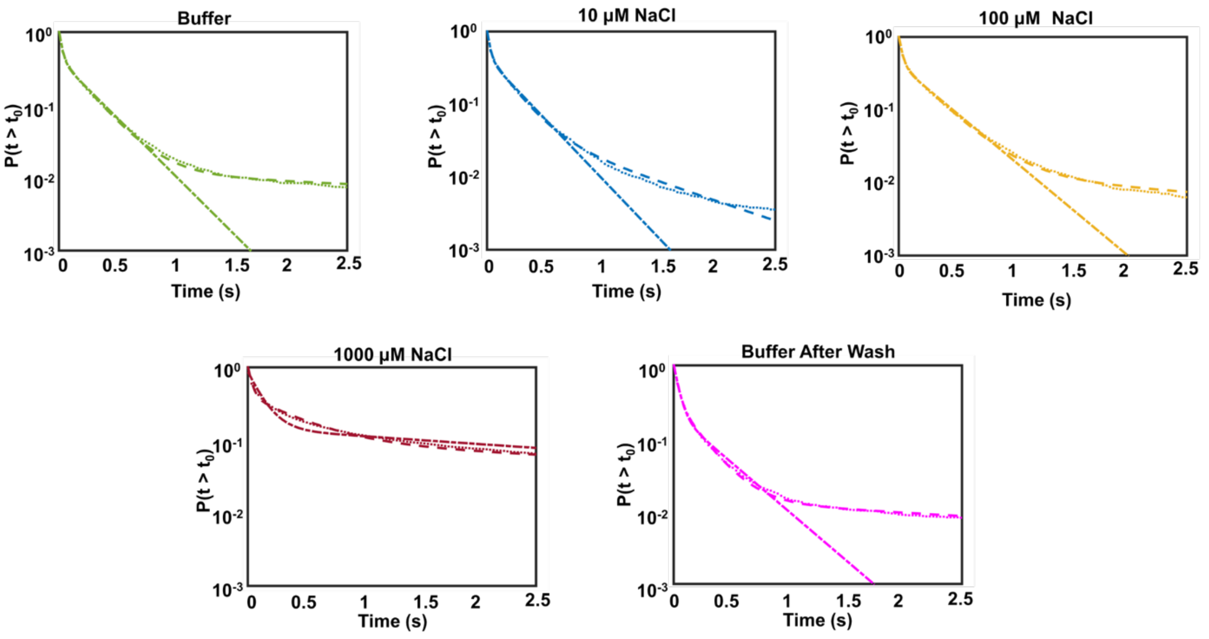

Figure S4: Cumulative distribution of SRTs for PS particles in buffer solution (green), 10 μM salt (blue), 100 μM salt (yellow), 1000 μM salt (dark red), and after washing with buffer from exposure to 1000 μM salt (pink). Three and two component fittings are represented as dashed and dashed-dotted lines, respectively. Three exponential fit provides a better fit.

SRT fitting parameters

	Buffer Only		10 µM		100 µM	
	Average	Standard Deviation	Average	Standard Deviation	Average	Standard Deviation
**b1**	0.433	0.017	0.438	0.008	0.370	0.087
**1/t1**	0.229	0.003	0.235	0.042	0.161	0.061
**b2**	0.010	0.004	0.061	0.050	0.177	0.121
**1/t2**	6.030	0.134	1.735	1.708	0.667	0.404
**b3**	0.556	0.021	0.500	0.044	0.453	0.035
**1/t3**	0.023	0.000	0.023	0.002	0.019	0.002
**tAvg**	0.172	0.025	0.172	0.016	0.155	0.028

	1000 µM		Buffer After Wash	
	Average	Standard Deviation	Average	Standard Deviation
**b1**	0.339	0.028	0.396	0.085
**1/t1**	0.353	0.041	0.224	0.031
**b2**	0.072	0.011	0.029	0.019
**1/t2**	6.626	1.001	4.826	0.696
**b3**	0.580	0.029	0.572	0.104
**1/t3**	0.037	0.004	0.035	0.006
**tAvg**	0.617	0.089	0.244	0.098

Table S3: Three component fitting parameters for SRT graphs for particles in buffer, 10 µM NaCl, 100 µM NaCl, and 1000 µM NaCl, and washing in buffer after experiment.

.

van-Hove correlation analysis

The distribution of particle displacements was quantified using the self-part of the van Hove correlation function,[6]

$$G_s(\Delta x, \Delta t) = \frac{1}{N}\langle \sum_{i=1}^{N} \delta(x + x_i(t) - x_i(t + \Delta t))\rangle \qquad (1)$$

where δ is the Dirac delta function, Δx is the displacement of all particles at a time of Δt, and the angle brackets denote an ensemble average. The van Hove function was estimated with a normalized histogram of one hundred bins binning all particle displacements.

The van Hove correlation distribution in all our datasets are separated into two parts: a center peak region around $\Delta x = 0$, and a broad "tail" region. These two regions of the van Hove correlation function can be fitted as two Gaussians, where both large and small displacements are common, or in cases where large displacements are rare, a Gaussian head with an exponential tail.[7]

Time dependent van-Hove graphs.

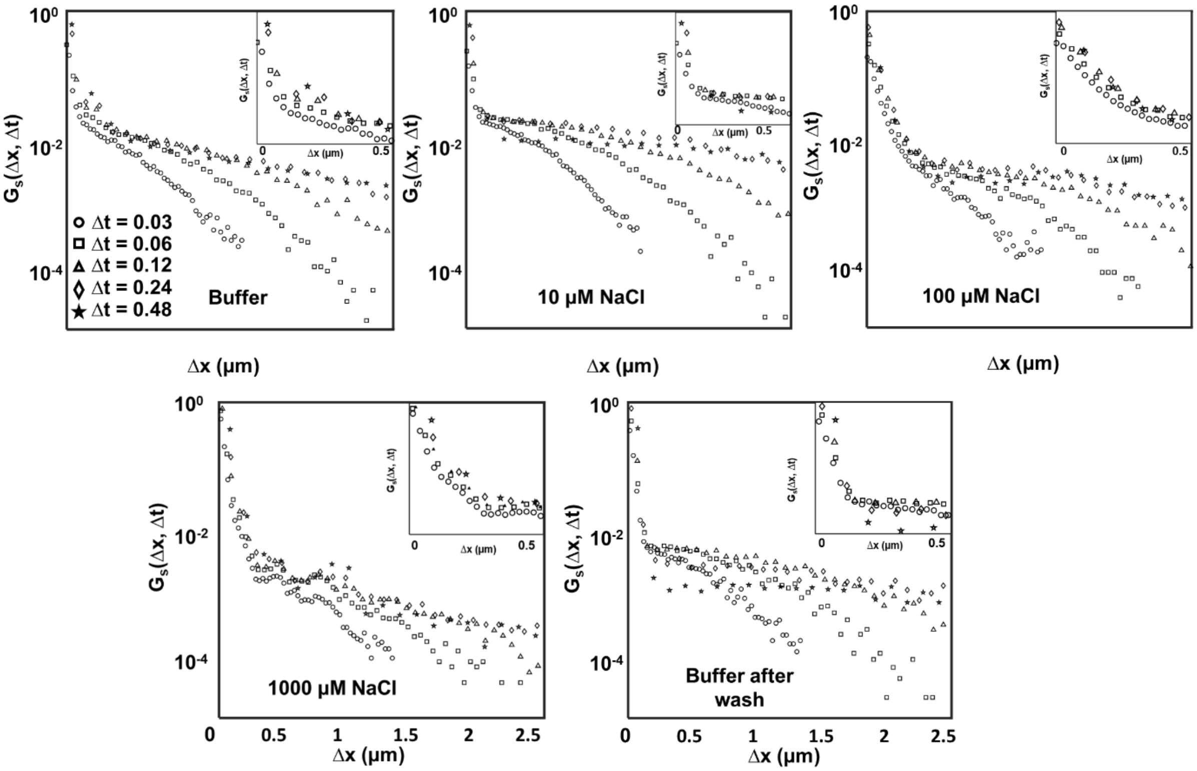

Figure S5: Time dependent van Hove graphs for particles in buffer, 10 μM salt, 100 μM salt, 1000 μM salt, and after washing with buffer. The insets shows a zoomed in section for the short displacements.

Non-Gaussian parameter analysis

The gaussianity of the van Hove correlation graphs were quantified through the calculation of the non-Gaussian parameter,[8]

$$\alpha_2(t) = \left|\frac{\langle \Delta x(\tau)^4 \rangle}{3\langle \Delta x(\tau)^2 \rangle^2} - 1\right| \quad (2)$$

where $\Delta x(\tau)$ is the displacement of all particles at a time of *t*. An α_2 value of zero represents Gaussianity, while a value greater than zero represents non-Gaussianity. The magnitude of α_2

signifies the non-Gaussianity of the van Hove graph, with larger α_2 values meaning greater non-Gaussian behavior in particle transport.

	10 µM		100 µM		1000 µM	
τ (frames)	Average α2	Standard Deviation α2	Average α2	Standard Deviation α2	Average α2	Standard Deviation α2
1	0.4103	0.4179	0.4337	0.4312	0.6117	1.4518
2	0.4022	0.4020	0.4161	0.3473	0.6405	1.6184
4	0.4014	0.3681	0.3994	0.3520	0.6802	1.7615
8	0.4199	0.3787	0.3945	0.3575	0.7363	1.8958
16	0.4596	0.5516	0.3915	0.4490	0.8076	2.1097

Table S4: Average and standard deviation of α_2 values of particles exposed to 10 µM, 100 µM, and 1000 µM salt.

Localization precision of our analysis

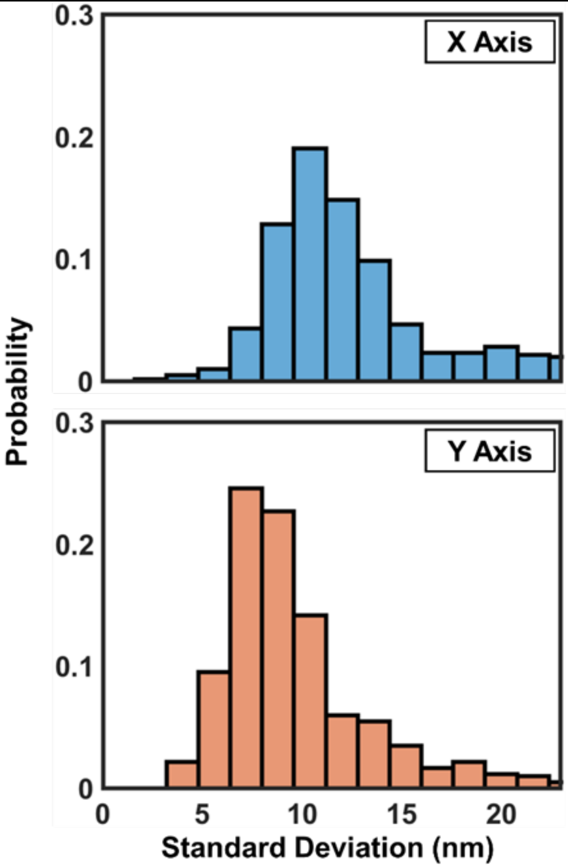

Figure S6: Localization precision of the X and Y axis of our TIRF microscope, calculated from localizing stationary beads using the same algorithm.

Mean square displacement (MSD) graphs and tables:

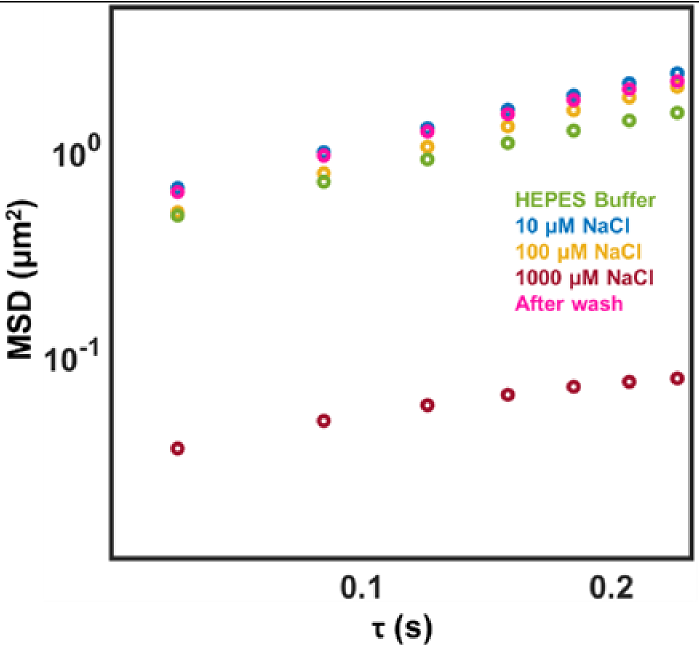

Figure S7: Average MSD of particles in buffer solution (green), 10 µM salt (blue), 100 µM salt (yellow), 1000 µM salt (brown), and after washing with buffer from exposure to 1000 µM salt (pink).

	Average ($\mu m^2 s^{-1}$)	Standard Deviation
**No SLB**	3.2	1.83
**Buffer**	3.26	1.84
**10 µM**	3.28	1.78
**100 µM**	1.84	2.19
**1000 µM**	1.03	1.72
**Buffer After Wash**	3.08	2.25

Table S5: Average diffusion coefficients of PS under different conditions

SLB membrane imaging with Nile Red (NR) dye

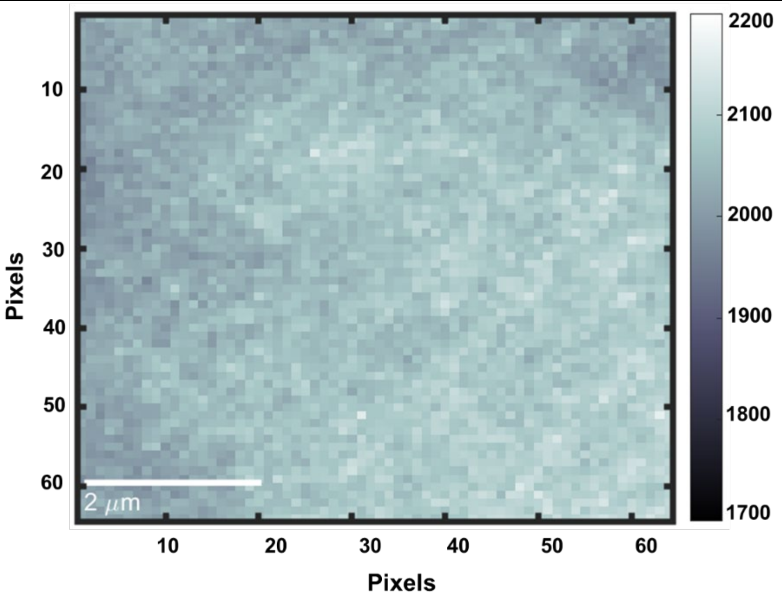

Figure S8: Uniform coverage of NR on SLB, representing the formation of a homogeneous single layer of lipid bilayer on plasma-cleaned coverslip.

SLB membrane imaging after salt exposure to the membrane

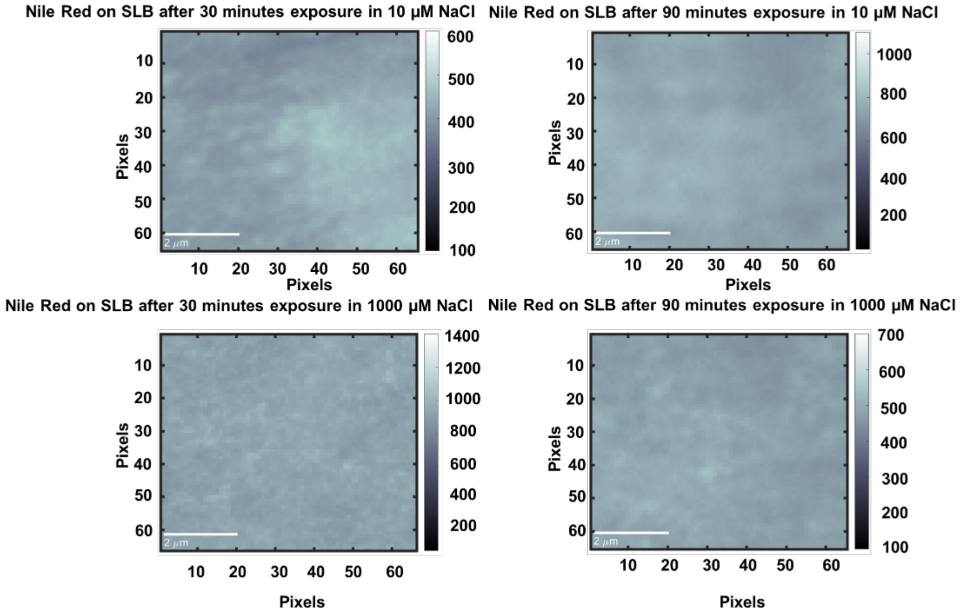

Figure S9: The surface coverage of NR on the SLB remained unchanged after exposure to salt for both 30 and 90 minutes, indicating that NaCl did not affect the coverage of the SLB.

System Preparations for molecular dynamics (MD) simulations

A polystyrene molecule comprising a linear chain of 25 styrene molecules (PS) immersed in water and ions was built using CHARMM-GUI.[9] A 400 ns of MD simulations were carried out for this system. Similarly, a POPC membrane (in water and ions) was built using CHARMM-GUI. Each leaflet of the membrane contains 200 POPC molecules. This system was equilibrated for 200 ns. The last structure of PS derived from the 400 ns simulation of PS in water was inserted into the last structure from the 200 ns simulations of the POPC system, resulting in the POPC + PS complex. The final system consists of POPC, PS, water, and ions. PS was initially positioned away from the membrane surface, and some water molecules were removed to make room for the PS

molecule. The concentration of NaCl in the system was kept at 0.1 M, representing a high salt concentration.

Details of the MD simulations

All MD simulations were performed using GROMACS[10] 2021 program with CHARMM36[11] force field. TIP3P[12] water model was used in all simulations. The cutoff distance for the van der Waals and the coulombic interactions was 1.2 nm. The particle mesh Ewald summation (PME)[13] method was used to calculate the long-range electrostatic interactions. A 5000 step of energy minimization was done using the steepest descent algorithm to minimize the system. Subsequently, the system was equilibrated for 1 ns under the NPT ensemble conditions, employing a semi-isotropic Parrinello-Rahman barostat[14, 15] set to maintain a pressure of 1 bar. The barostat was configured with a coupling constant of 5 ps and a compressibility of 4.5 × 10−5 bar−1, applied in both the lateral and membrane normal directions. Nose-Hoover thermostat[16, 17] was used to maintain the temperature at 310 K. A production simulation of 1 μs was carried out from the last step of the equilibration. Only the last 800 ns simulations from the production run are used for analysis.

We also performed steered molecular dynamics (SMD) simulations to pull polystyrene from the bulk water to the center of the membrane. The center of mass of the PS was subjected to an external force with a spring constant of 1000 kJ mol^{-1} nm^{-2}, facilitating its movement across the membrane at a constant velocity of 0.1 nm ns^{-1}. The lateral motion of the PS was not constrained. A constraint was applied to the phosphorous atoms of the membrane during the SMD simulations.

Calculation of potential of mean force (PMF)

We calculated the PMF as a function of the distance between the center of mass (COM) of PS and COM of the membrane, using Umbrella Sampling (US)[18] technique and the weighted histogram analysis method (WHAM)[19] implemented in GROMACS. First, initial conformations were stored at intervals of approximately 0.1 nm along the SMD simulation trajectory. They were subsequently used to initiate independent MD simulations in each umbrella sampling window for 100 ns. The center of mass varies within a Δz-wide sampling window, while the z-location of the PS was fixed in each window. Finally, the PMF was obtained by integrating the force over z after it had been averaged over time and distance in each window.

We calculate the PMF of PS as a function of the distance (Δz) between COM of PS and COM of POPC, shown in Fig. S1(a). We set the minimum PMF value for PS in bulk water as the reference (PMF = 0). The PMF shows a peak at Δz = 17.4 Å. The barrier height, defined as the PMF difference between the peak and the tail of the PMF profile, is observed to be ~29.5 kJ/mol. The PMF shows that PS is highly stable inside the POPC due to the hydrophobic interactions between the PS and the lipid tails. We show three snapshots of the systems, one at R1, another at R2, and the third at R3, shown in Figs. S1 (b)-(d), respectively.

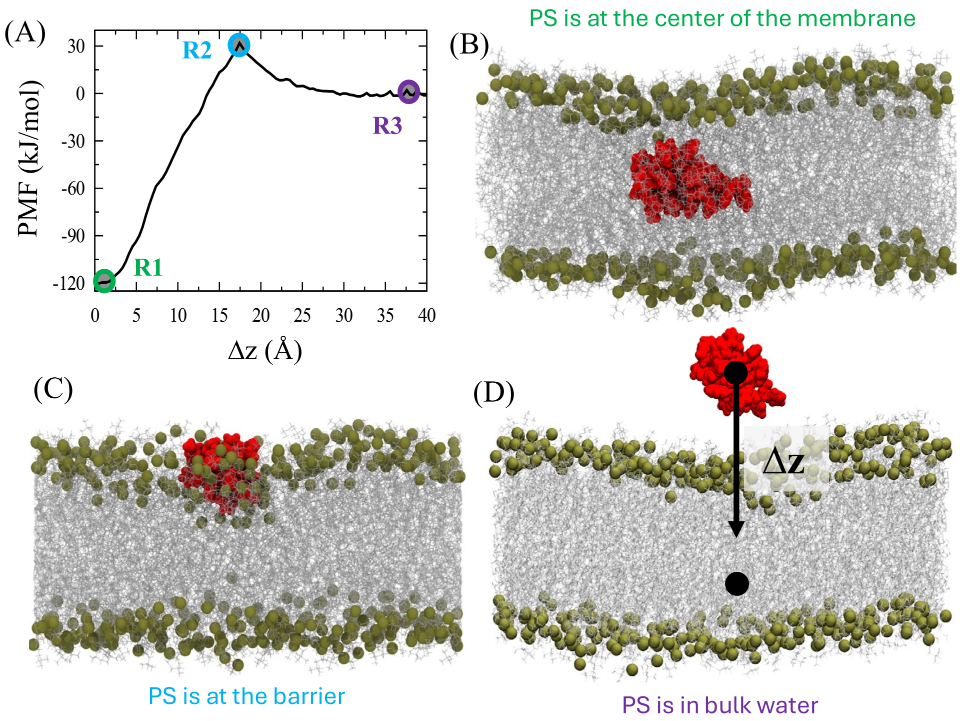

Fig. S10. (A) PMF of PS insertion as a function of the distance (Δz) between the COM of PS and COM of POPC. Three different regions are marked in circles in the PMF profile: one at the minimum of the PMF (inside POPC), shown in green circle (R1), another at the barrier, shown in cyan circle (R2), and the third at the tail (bulk water), shown in purple circle (R3). The Snapshots corresponding to these three regions are shown in Figs. (B)-(D). Water molecules are not shown due to clarity. Polystyrene is illustrated in red with vdw representation. The lipids are shown in grey with stick representation, and phosphorous atoms are shown in tan spheres.

Analysis

Calculation of number density

We calculate the number density, which is defined by $\frac{n}{V}$. Here, n is the number of atoms, and V is the box volume corresponding to a particular bin of 1 Å width. The density is calculated along the z direction of the box. We calculate the number density of PO_4^-, NMe_3^+, Na^+, and Cl^- ions from the last 800 ns of the equilibration MD simulations. We also calculate the number density for the

same headgroups and ions from the three different umbrella sampling windows. The three windows represent three different regions in the PMF: one chosen near the minimum (R1), one at the barrier (R2), and one from the tail (R3) of the PMF. Note that the PS is in the bulk water at the R3 window.

Table S6. Average number of Na^+ ions present within 5.0 A of PO_4^- headgroup, and the number of Cl^- ions present within 5.0 Å of NMe_3^+ headgroup. This number is averaged over the corresponding simulations.

Simulations	$Na^+ - PO_4^-$	$Cl^- - NMe_3^+$
Equilibrium simulation (800 ns)	13.52	5.01
PS at R1 (100 ns)	14.12	5.26
PS at R2 (100 ns)	13.47	5.14
PS at R3 (100 ns)	14.98	5.39

We further extend our analysis by computing density profiles for PO_4^- and NMe_3^+ ions, as well as sodium and chloride ions, along the z-axis of the simulation box across three distinct umbrella sampling windows, R1 (Fig. S11A and S11B), R2 (Fig. S11C and S11D), and R3 (Fig. S11E and S11F). The density profiles for the headgroups and the ions do not show any significant difference between any of the windows compared to the density profiles obtained from equilibrium

simulations. This suggests that neither the position nor the peak of the density profiles for the ions or the headgroups is impacted by the location of PS.

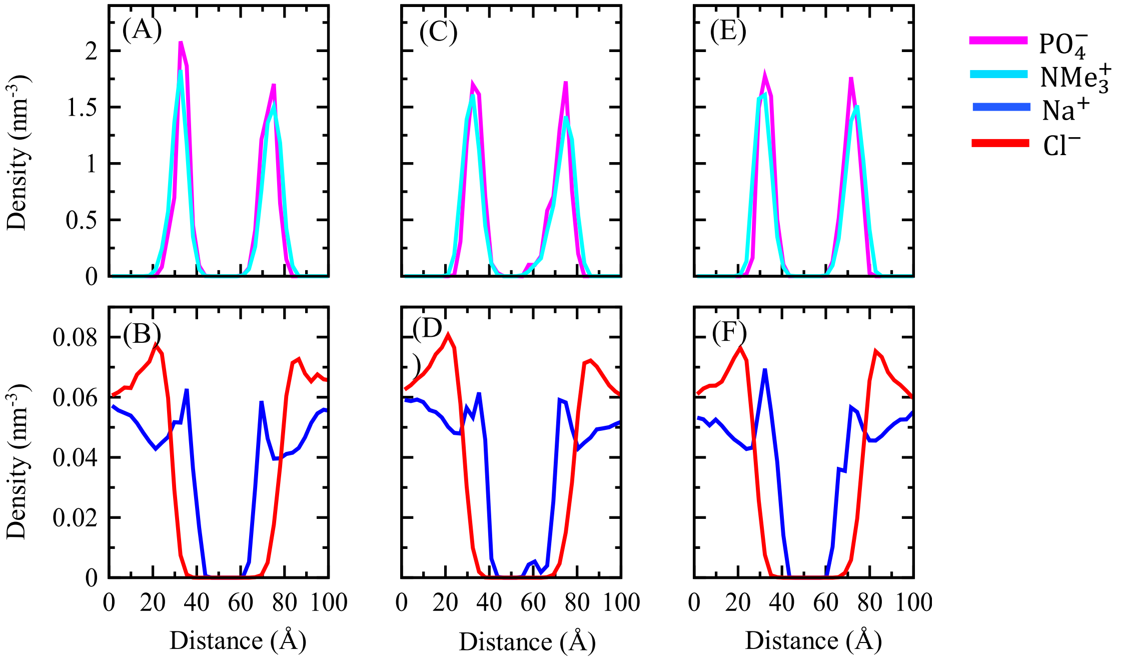

Fig. S11. Density profiles of PO_4^- and NMe_3^+(cyan) from 100 ns simulation in the umbrella sampling corresponding to (A) R1, (C) R2, and (E) R3 windows in Figure S10A. Density profiles of Na^+ and Cl^- from 100 ns simulation in the umbrella sampling corresponding to (B) R1, (D) R2, and (F) R3 windows in Figure S10A.

References:

(1) Lind, T. K.; Cárdenas, M.; Wacklin, H. P. Formation of supported lipid bilayers by vesicle fusion: effect of deposition temperature. *Langmuir* **2014**, *30* (25), 7259-7263.

(2) Johnson, J. M.; Ha, T.; Chu, S.; Boxer, S. G. Early steps of supported bilayer formation probed by single vesicle fluorescence assays. *Biophys. J.* **2002**, *83* (6), 3371-3379.

(3) Shuang, B.; Chen, J.; Kisley, L.; Landes, C. F. Troika of single particle tracking programing: SNR enhancement, particle identification, and mapping. *Phys. Chem. Chem. Phys.* **2014**, *16* (2), 624-634.

(4) Parthasarathy, R. Rapid, accurate particle tracking by calculation of radial symmetry centers. *Nat. Methods* **2012**, *9* (7), 724-726.

(5) Tauzin, L. J.; Shuang, B.; Kisley, L.; Mansur, A. P.; Chen, J.; de Leon, A.; Advincula, R. C.; Landes, C. F. Charge-dependent transport switching of single molecular ions in a weak polyelectrolyte multilayer. *Langmuir* **2014**, *30* (28), 8391-8399.

(6) Skaug, M. J.; Mabry, J.; Schwartz, D. K. Intermittent molecular hopping at the solid-liquid interface. *Phys. Rev. Lett.* **2013**, *110* (25), 256101.

(7) Wang, B.; Kuo, J.; Bae, S. C.; Granick, S. When Brownian diffusion is not Gaussian. *Nat. Mater.* **2012**, *11* (6), 481-485.

(8) Xu, C.; Yang, K.; Yuan, B. Non-Gaussian diffusion of individual lipids unveils the unique peptide–membrane interaction dynamics. *J. Phys. Chem. Lett.* **2023**, *14* (4), 854-862.

(9) Jo, S.; Kim, T.; Iyer, V. G.; Im, W. CHARMM-GUI: a web-based graphical user interface for CHARMM. *J. Comput. Chem.* **2008**, *29* (11), 1859-1865.

(10) Berendsen, H. J.; van der Spoel, D.; van Drunen, R. GROMACS: A message-passing parallel molecular dynamics implementation. *Comput. Phys. Commun.* **1995**, *91* (1-3), 43-56.

(11) Klauda, J. B.; Venable, R. M.; Freites, J. A.; O'Connor, J. W.; Tobias, D. J.; Mondragon-Ramirez, C.; Vorobyov, I.; MacKerell Jr, A. D.; Pastor, R. W. Update of the CHARMM all-atom additive force field for lipids: validation on six lipid types. *J. Phys. Chem. B* **2010**, *114* (23), 7830-7843.

(12) Jorgensen, W. L.; Chandrasekhar, J.; Madura, J. D.; Impey, R. W.; Klein, M. L. Comparison of simple potential functions for simulating liquid water. *J. Chem. Phys.* **1983**, *79* (2), 926-935.

(13) Darden, T.; York, D.; Pedersen, L. Particle mesh Ewald: An N· log (N) method for Ewald sums in large systems. *J. Chem. Phys.* **1993**, *98* (12), 10089-10092.

(14) Parrinello, M.; Rahman, A. Polymorphic transitions in single crystals: A new molecular dynamics method. *J. Appl. Phys.* **1981**, *52* (12), 7182-7190.

(15) Nosé, S.; Klein, M. Constant pressure molecular dynamics for molecular systems. *Mol. Phys.* **1983**, *50* (5), 1055-1076.

(16) Nosé, S. A unified formulation of the constant temperature molecular dynamics methods. *J. Chem. Phys.* **1984**, *81* (1), 511-519.

(17) Hoover, W. G. Canonical dynamics: Equilibrium phase-space distributions. *Physical review A* **1985**, *31* (3), 1695.

(18) Torrie, G. M.; Valleau, J. P. Nonphysical sampling distributions in Monte Carlo free-energy estimation: Umbrella sampling. *J. Comput. Phys.* **1977**, *23* (2), 187-199.

(19) Kumar, S.; Rosenberg, J. M.; Bouzida, D.; Swendsen, R. H.; Kollman, P. A. The weighted histogram analysis method for free-energy calculations on biomolecules. I. The method. *J. Comput. Chem.* **1992**, *13* (8), 1011-1021.
